# Gender differences in the relationship between cardiometabolic index and all-cause and specific mortality in the United States adults: a national study

**DOI:** 10.3389/fendo.2025.1525815

**Published:** 2025-02-17

**Authors:** Tianshu Li, Haoran Zhou, Hua Zhou

**Affiliations:** ^1^ Institute of Cardiovascular Disease of Integrated Traditional Chinese and Western Medicine, Shuguang Hospital Affiliated to Shanghai University of Traditional Chinese Medicine, Shanghai, China; ^2^ Branch of National Clinical Research Center for Chinese Medicine Cardiology, Shuguang Hospital Affiliated to Shanghai University of Traditional Chinese Medicine, Shanghai, China; ^3^ First Clinical Medical College, Shandong University of Traditional Chinese Medicine, Jinan, China; ^4^ Department of Cardiology, Shuguang Hospital Affiliated to Shanghai University of Traditional Chinese Medicine, Shanghai, China

**Keywords:** cardiometabolic index, all-cause mortality, specific mortality, gender differences, NHANES

## Abstract

**Background:**

The cardiometabolic index (CMI) is a new comprehensive measure that reflects visceral obesity and metabolic function. This study aimed to examine associations between CMI and adult mortality from all causes and specific causes, as well as gender differences, using the National Health and Nutrition Examination Survey (NHANES) database.

**Methods:**

We included 37,539 adult participants with complete data from the 1999-2018 NHANES database. We categorized the participants according to gender and constructed three models to investigate the relationship between CMI and the outcome variables. These were analyzed using Kaplan-Meier curve analysis, COX proportional risk models, and restricted cubic spline (RCS).

**Results:**

Baseline characteristics showed that among both male and female participants, those who died exhibited higher levels of CMI compared to those who survived. Kaplan-Meier curves showed an increasing trend in all-cause and specific mortality with increasing follow-up time. When CMI was categorized according to quartiles (Q1-Q4), the probability of survival was lower in the Q4 group compared to Q1. We found no gender differences between all three mortality rates. In COX regression analyses, all-cause, cardiovascular, and diabetes mortality were significantly higher in Q4 in the whole population and female participants, whereas no significant differences were identified among male participants. The RCS showed a nonlinear positive correlation in diabetes mortality for females and a linear positive correlation in all-cause and cardiovascular mortality. As for males, CMI was positively and nonlinearly associated with all-cause and diabetes mortality. Besides, there is no statistically significant correlation for males in cardiovascular mortality.

**Conclusion:**

There were gender differences in the correlation between CMI and all-cause mortality, cardiovascular mortality, and diabetes mortality in the adult population. The findings indicated that adult females with elevated CMI levels were at an elevated risk of mortality from all causes, cardiovascular disease, and diabetes. At the same time, there were no significant associations in adult males.

## Graphical Abstract

Overview of the methodology and main results of the study of gender differences in the association between CMI and all-cause and specific mortality. Red arrows indicate statistically significant elevations. White circles indicate no statistical significance. Materials provided by FigDraw (www.figdraw.com).

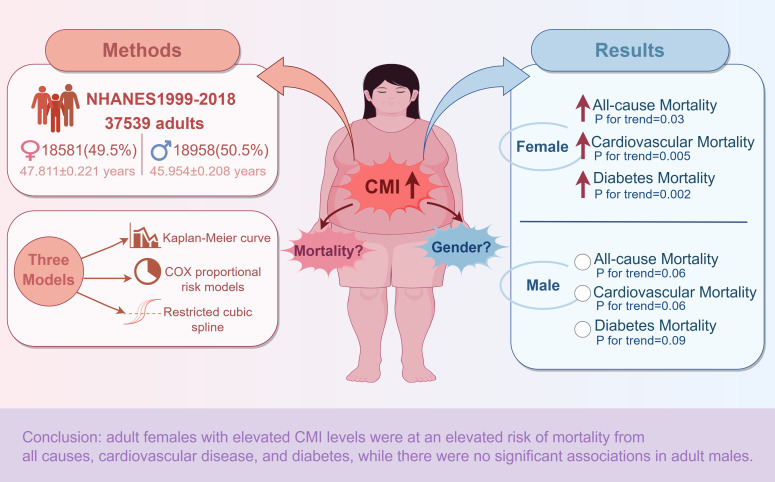

## Introduction

Cardiometabolic diseases are a series of metabolic dysfunctions, including cardiovascular diseases and diabetes mellitus. Among adults worldwide, they constitute one of the primary causes of morbidity and mortality (1, 2). Effective identification and active control of cardiometabolic diseases in their early stages represent a crucial determinant of disease development and prognosis. The development of cardiometabolic diseases is most closely related to obesity (3, 4). In the past, we usually used body mass index (BMI) to define obesity (5). However, this approach cannot determine the proportion of fat in the body composition and thus has many limitations (6). In recent years, a growing number of people with normal-weight obesity (NWO) has prompted a surge in research interest in the health implications of visceral obesity (7).

Visceral obesity is directly related to cardiometabolic diseases (8), and many new measures have emerged for assessing visceral obesity. Wakabayashi et al. (9) were the first to propose CMI, which is derived from a combination of lipids and obesity parameters and can be calculated to detect cardiometabolic risk in humans simply and effectively, providing a comprehensive assessment of cardiometabolic risk (10–12).

In recent years, relevant studies have found that CMI may be a marker for predicting renal function (13), depression (14), impaired fasting glucose, insulin resistance, type 2 diabetes mellitus (15), non-alcoholic fatty liver disease and fibrosis (16), biological aging (17), endometriosis (18, 19), and erectile dysfunction (20) in older adults. Regarding the effect of CMI on mortality, recent studies have shown that elevated CMI is positively associated with all-cause mortality in middle-aged and older adults and is partially mediated by inflammation (21, 22). Moreover, in the general population, CMI has an L-shaped nonlinear association with all-cause mortality (23).

Based on the above research, we have three research gaps. Firstly, we focused on adults over 18, the leading group who develop cardiometabolic diseases. Secondly, because CMI is associated with metabolic diseases, we focused on the correlation between CMI and all-cause, cardiovascular, and diabetes mortality. To our knowledge, this is the first study to include the correlation between CMI and diabetes mortality. Finally, there are differences in whole-body lipid metabolism between men and women, and trends in body fat accumulation in women are associated with an increased risk of metabolic-related diseases (24), so we further explored sex differences between CMI and different mortality correlations.

## Methods

### Research population

A cross-sectional survey of U.S. citizens’ health and nutritional status, NHANES, was conducted in 1999 and contains a wide range of demographic, economic, nutritional, and health data (25). NHANES employs a sophisticated, four-stage sampling design, utilizing a stratified, multistage probability sampling approach to ensure the sample is representative of the entire nation (26). Fifteen different sites are selected each year from a sampling frame of all U.S. counties, from which it chooses a nationally representative sample of comprising approximately 5,000 individuals (27). Written informed consent and ethical review by the National Centre for Health Statistics (NCHS) Ethics Review Committee were obtained from all participants in the NHANES study.

**Figure 1:**
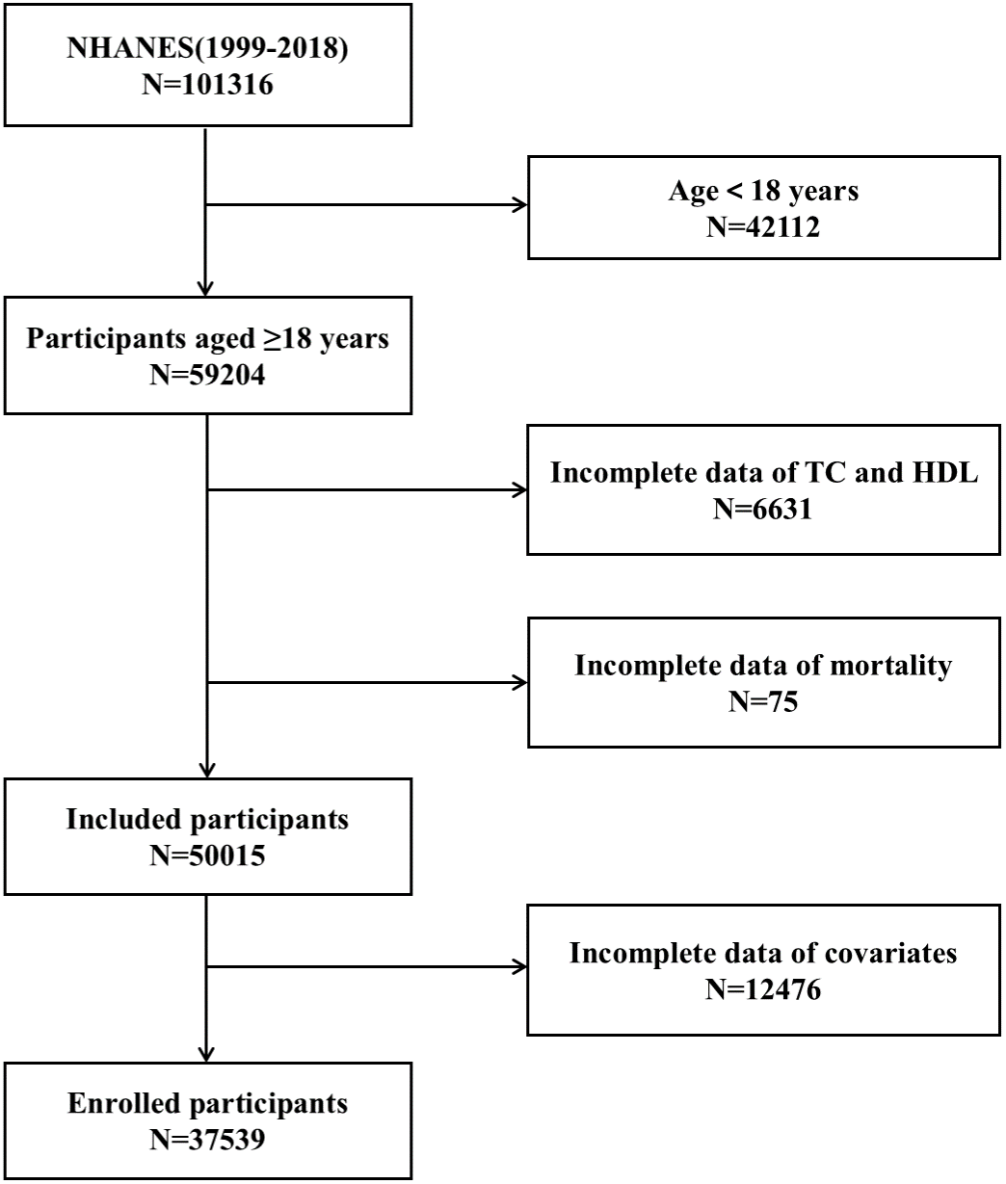
Flowchart for inclusion in the study population.

In this study, we selected 101,316 participants who participated in the NHANES survey from 1999 to 2018 and linked them to NDI data to obtain information on participant follow-up. After excluding those who failed to meet the inclusion criteria, the study finally included 37539 participants. The flowchart in **Figure 1** illustrates the process for screening the inclusion population.

### Definition of CMI

CMI, as an exposure variable in this study, was derived by calculation from blood indicators and body data measured by professionals in a mobile examination van. The formula is as follows (9): CMI=TG(mg/dl)/HDL(mg/dl)×Waist(cm)/Height(cm).

### Determination of outcome variables

The outcome variables in this research included all-cause mortality and two specific mortality rates. The specific mortality rates included cardiovascular mortality and diabetes mortality. We obtained mortality-related data from the NDI Death Certificate Record (www.cdc.gov/nchs/data-linkage/mortality-public.htm), which is currently updated until December 31, 2019 (28). Combining NHANES and NDI-related data provides comprehensive information on participant deaths, including time of death and cause of death, among other comprehensive information. Based on the fact that the study period is 1999-2018, we used the person-months from the date of interview to the date of death or the end of the period of death (PERMTH_INT) to calculate the follow-up time in this study. People can obtain specific information from the official website mentioned above. Specifically, the codes I00-09, I11, I13, I20-51, and I60-I69 identify cardiovascular mortality, while the codes E10-E14 identify diabetes mortality in the International Classification of Diseases (ICD)-10.

### Assessment of covariates

The covariates in the study consisted primarily of demographic characteristics of gender (male, female), age (years), race (Mexican American, non-Hispanic white, non-Hispanic black, other Hispanic, and other race), marriage (married, never married, separated, divorced, widowed, and living with partner), household income (Poverty Income Ratio, PIR), and educational qualification (less than 9th grade, 9-11th grade, high school graduate, some college, and college graduate or above). Other covariates included body mass index (BMI, kg/m^2^), alcohol intake (never, light/moderate, heavy, former), and smoking status (never, current, former). Laboratory tests included triglycerides (TG, mg/dl), total cholesterol (TC, mg/dl), high-density lipoprotein (HDL, mg/dl), and glomerular filtration rate (eGFR, mL/min/1.73 m^2^). A questionnaire determined coronary heart disease (CHD) diagnosis based on self-reported responses. A diagnosis of diabetes mellitus(DM) is the fulfillment of any of the following conditions: (1) doctor told you have diabetes, (2) glycohemoglobin HbA1c (%) >= 6.5, (3) fasting glucose (mmol/l) >= 7.0, (4) random blood glucose (mmol/l) >= 11.1, (5) two-hour OGTT blood glucose (mmol/l) >= 11.1, (6) Use of diabetes medication or insulin. The diagnosis of hypertension is based on the following criteria: (1) self-reported hypertension, (2) mean systolic blood pressure (SBP) ≥140 mmHg and/or mean diastolic blood pressure (DBP) ≥90 mmHg, and (3) use of antihypertensive medications. The diagnosis of hyperlipidemia is fulfilling the following conditions: (1) diagnosis of hypertriglyceridemia (TG>=150mg/dL), (2) diagnosis of hypercholesterolemia: (i) TC>=200mg/dL, (ii) low-density lipoprotein cholesterol (LDL-C) >=130mg/dL (iii) HDL<40mg/dL in men and <50mg/dL in women, and (3) current taking lipid-lowering drugs. The official NHANES website provides specific technical information regarding covariate determination.

### Statistical analyses

Every analysis in this research utilized the R language (version 4.3.0) and achieved statistical significance with a two-tailed p-value below 0.05. We divided participants into four groups according to their CMI quartiles (Q1-Q4), and the lowest quartile was considered the baseline. We divided the population based on gender and CMI quartiles to investigate the relationship between CMI and all-cause and specific mortality. The weights of the analyses were adjusted to prevent oversampling. We used weighted means (95% confidence intervals [CIs]) and weighted percentages (95% confidence intervals) to describe continuous and categorical variables, respectively (29). To assess gender differences in the relationship between CMI and outcome variables, we devised three weighted COX regression models, with model 1 unadjusted for confounders, model 2 adjusted for age and ethnicity, and model 3 adjusted for age, race, marital status, smoking status, alcohol use, eGFR, and TC.

In addition, the RCS was employed with three nodes (5th, 50th, and 95th percentiles) to assess dose-response patterns between CMI and all-cause and specific mortality. We generated Kaplan-Meier survival curves for various mortality rates based on CMI quartiles and survival times.

## Results

### Baseline characteristics

We included 37,539 participants over ten consecutive survey cycles from 1999 to 2018. **Tables 1**, **2** demonstrate the baseline characteristics of participants by CMI quartile (Q1-Q4) by gender, with a mean age of 45.954 for male participants and 47.811 for female participants. In Q1-Q4, there were notable disparities (p < 0.05) between males and females in age, gender, race, marriage, education, poverty level, BMI, smoking, alcohol consumption, CHD, DM, hypertension, hyperlipidemia, height, waist circumference, total cholesterol, triglycerides, HDL, and glomerular filtration rate. **Supplementary Table S1** demonstrates the baseline characteristics of participants by survival status and shows that those who died exhibited higher levels of CMI compared with those who survived.

**Table 1:** Baseline characteristics of male participants.

Variables	CMI					P-value
	Total (n=18958)	Q1 (n=3544)	Q2 (n=4371)	Q3 (n=4957)	Q4 (n=6086)	
CMI	2.552 ± 0.034	0.508 ± 0.003	1.015 ± 0.003	1.836 ± 0.006	5.384 ± 0.075	< 0.0001
Age (years)	45.954 ± 0.208	42.346 ± 0.414	45.561 ± 0.377	47.015 ± 0.283	47.442 ± 0.260	< 0.0001
Race, n (%)						< 0.0001
Mexican American	3279 (8.545%)	390 (5.896%)	620 (7.118%)	917 (9.189%)	1352 (10.559%)	
Non-Hispanic Black	3695 (9.362%)	1160 (16.559%)	1056 (11.331%)	825 (7.801%)	654 (5.102%)	
Non-Hispanic White	8995 (70.812%)	1533 (68.038%)	2028 (70.809%)	2416 (71.204%)	3018 (72.079%)	
Other Race	1604 (6.167%)	287 (5.691%)	379 (5.992%)	410 (6.289%)	528 (6.467%)	
Others Hispanic	1385 (5.114%)	174 (3.816%)	288 (4.749%)	389 (5.516%)	534 (5.794%)	
Marital status, n (%)						< 0.0001
Divorced	1630 (8.161%)	286 (7.668%)	352 (7.437%)	444 (8.194%)	548 (8.932%)	
Living with partner	1551 (8.213%)	338 (9.996%)	315 (7.316%)	390 (8.395%)	508 (7.695%)	
Married	11123 (60.260%)	1643 (49.956%)	2512 (58.399%)	3043 (61.887%)	3925 (66.158%)	
Never married	3408 (19.173%)	1004 (28.048%)	895 (22.529%)	765 (17.114%)	744 (13.365%)	
Separated	497 (1.992%)	112 (2.003%)	113 (2.095%)	127 (2.228%)	145 (1.725%)	
Widowed	749 (2.201%)	161 (2.329%)	184 (2.224%)	188 (2.183%)	216 (2.124%)	
Education, n (%)						< 0.0001
Less than 9th grade	2188 (5.496%)	291 (4.179%)	438 (5.001%)	593 (5.772%)	866 (6.380%)	
9-11th grade	2803 (11.109%)	563 (11.622%)	617 (9.925%)	723 (11.334%)	900 (11.484%)	
High school graduate	4542 (24.870%)	852 (23.781%)	1025 (23.796%)	1174 (24.385%)	1491 (26.644%)	
Some College	5089 (29.470%)	952 (26.983%)	1171 (29.545%)	1306 (29.351%)	1660 (30.928%)	
College graduate or above	4336 (29.054%)	886 (33.435%)	1120 (31.733%)	1161 (29.159%)	1169 (24.564%)	
PIR						0.013
<1.30	5299 (18.447%)	1019 (19.117%)	1126 (17.275%)	1306 (17.712%)	1848 (19.490%)	
1.30-3.50	7237 (35.154%)	1343 (35.447%)	1625 (33.934%)	1930 (34.663%)	2339 (36.251%)	
≥3.50	6422 (46.398%)	1182 (45.437%)	1620 (48.791%)	1721 (47.625%)	1899 (44.259%)	
BMI, kg/m^2^						< 0.0001
<25	5290 (26.912%)	2144 (59.391%)	1564 (35.541%)	991 (19.290%)	591 (8.335%)	
25-30	7398 (39.020%)	1094 (32.507%)	1831 (42.516%)	2160 (44.447%)	2313 (35.906%)	
≥30	6270 (34.068%)	306 (8.103%)	976 (21.944%)	1806 (36.263%)	3182 (55.759%)	
Smoking status, n (%)						< 0.0001
Former	5854 (29.180%)	852 (23.921%)	1305 (27.985%)	1649 (31.389%)	2048 (31.266%)	
Never	8422 (46.806%)	1703 (49.887%)	2021 (49.057%)	2124 (44.916%)	2574 (44.950%)	
Now	4682 (24.014%)	989 (26.192%)	1045 (22.959%)	1184 (23.695%)	1464 (23.784%)	
Alcohol use, n (%)						< 0.0001
Former	3362 (13.982%)	443 (9.116%)	708 (12.503%)	954 (14.913%)	1257 (17.066%)	
Heavy	4598 (25.507%)	885 (26.427%)	1038 (26.657%)	1154 (23.756%)	1521 (25.557%)	
Mild/moderate	9557 (54.001%)	1948 (58.328%)	2283 (54.223%)	2478 (55.131%)	2848 (50.480%)	
Never	1441 (6.510%)	268 (6.129%)	342 (6.616%)	371 (6.200%)	460 (6.898%)	
CHD, n (%)						< 0.0001
No	17878 (95.439%)	3422 (97.263%)	4142 (96.187%)	4634 (94.508%)	5680 (94.609%)	
Yes	1080 (4.561%)	122 (2.737%)	229 (3.813%)	323 (5.4925)	406 (5.391%)	
DM, n (%)						< 0.0001
No	15626 (87.068%)	3252 (94.608%)	3801 (91.371%)	4047 (87.304%)	4526 (79.512%)	
Yes	3332 (12.932%)	292 (5.392%)	570 (8.629%)	910 (12.696%)	1560 (20.488%)	
Hypertension, n (%)						< 0.0001
No	10972 (62.878%)	2456 (76.137%)	2660 (67.657%)	2751 (60.626%)	3105 (53.710%)	
Yes	7986 (37.122%)	1088 (23.863%)	1711 (32.343%)	2206 (39.374%)	2981 (46.290%)	
Hyperlipidemia, n (%)						< 0.0001
No	5491 (29.428%)	2151 (63.148%)	1836 (42.545%)	1187 (23.817%)	317 (5.334%)	
Yes	13467 (70.572%)	1393 (36.852%)	2535 (57.455%)	3770 (76.183%)	5769 (94.666%)	
Height, cm	176.120 ± 0.086	176.586 ± 0.173	176.362 ± 0.155	176.028 ± 0.132	175.754 ± 0.142	< 0.001
Waist, cm	101.356 ± 0.190	89.329 ± 0.256	97.198 ± 0.300	103.232 ± 0.265	109.676 ± 0.294	< 0.0001
TC, mg/dL	194.139 ± 0.483	179.781 ± 0.879	188.117 ± 0.822	194.144 ± 0.791	206.610 ± 0.821	< 0.0001
TG, mg/dL	168.892 ± 1.765	62.058 ± 0.389	96.192 ± 0.525	141.810 ± 0.733	303.216 ± 4.018	< 0.0001
HDL, mg/dL	47.648 ± 0.165	63.630 ± 0.357	52.084 ± 0.249	45.120 ± 0.160	37.397 ± 0.125	< 0.0001
eGFR, mL/min/1.73 m^2^	93.890 ± 0.259	98.519 ± 0.482	94.006 ± 0.414	92.535 ± 0.384	92.252 ± 0.370	< 0.0001

Continuous variables are expressed as weighted means ( ± SE) and categorical variables as unweighted frequencies (weighted percentages).

**Table 2:** Baseline characteristics of female participants.

Variables	CMI					P-value
	Total (n=18581)	Q1 (n=5310)	Q2 (n=4932)	Q3 (n=4517)	Q4 (n=3822)	
CMI	1.679 ± 0.022	0.484 ± 0.003	1.006 ± 0.003	1.802 ± 0.006	4.453 ± 0.070	< 0.0001
Age (years)	47.811 ± 0.221	43.512 ± 0.308	47.699 ± 0.354	50.748 ± 0.305	51.579 ± 0.345	< 0.0001
Race, n (%)						< 0.0001
Mexican American	3153 (6.948%)	528 (4.426%)	792 (6.805%)	931 (8.323%)	902 (9.676%)	
Non-Hispanic Black	3705 (10.571%)	1397 (13.282%)	1090 (11.468%)	817 (9.789%)	401 (5.764%)	
Non-Hispanic White	8619 (71.070%)	2518 (71.489%)	2273 (70.902%)	1965 (68.808%)	1863 (73.291%)	
Other Race	1540 (6.154%)	518 (6.426%)	379 (5.631%)	353 (6.651%)	290 (5.858%)	
Others Hispanic	1564 (5.257%)	349 (4.377%)	398 (5.194%)	451 (6.429%)	366 (5.411%)	
Marital status, n (%)						< 0.0001
Divorced	2395 (12.396%)	642 (11.380%)	620 (11.976%)	577 (12.355%)	556 (14.709%)	
Living with partner	1244 (6.789%)	385 (6.999%)	338 (7.038%)	271 (6.090%)	250 (6.917%)	
Married	8954 (54.088%)	2497 (54.247%)	2316 (53.040%)	2264 (55.584%)	1877 (53.539%)	
Never married	3031 (15.255%)	1193 (19.604%)	847 (16.053%)	581 (12.227%)	410 (10.547%)	
Separated	714 (2.769%)	165 (2.332%)	203 (2.910%)	181 (3.136%)	165 (2.856%)	
Widowed	2243 (8.703%)	428 (5.438%)	608 (8.983%)	643 (10.608%)	564 (11.432%)	
Education, n (%)						< 0.0001
Less than 9th grade	1864 (4.717%)	235 (2.160%)	498 (4.758%)	596 (6.464%)	535 (6.806%)	
9-11th grade	2567 (10.329%)	543 (6.911%)	632 (9.642%)	724 (12.463%)	668 (14.408%)	
High school graduate	4160 (22.936%)	1016 (18.134%)	1106 (23.247%)	1060 (24.982%)	978 (27.984%)	
Some College	5872 (33.483%)	1752 (32.600%)	1608 (34.591%)	1351 (32.705%)	1161 (34.293%)	
College graduate or above	4118 (28.534%)	1764 (40.196%)	1088 (27.762%)	786 (23.386%)	480 (16.509%)	
PIR						< 0.0001
<1.30	5797 (21.883%)	1284 (16.702%)	1477 (21.364%)	1531 (23.860%)	1505 (28.812%)	
1.30-3.50	7046 (36.000%)	1906 (32.537%)	1930 (36.542%)	1755 (38.261%)	1455 (38.265%)	
≥3.50	5738 (42.117%)	2120 (50.762%)	1525 (42.094%)	1231 (37.879%)	862 (32.923%)	
BMI, kg/m^2^						< 0.0001
<25	5782 (35.115%)	3144 (64.285%)	1548 (34.250%)	746 (17.645%)	344 (8.958%)	
25-30	5386 (28.059%)	1369 (24.127%)	1624 (33.763%)	1363 (29.311%)	1030 (24.993%)	
≥30	7413 (36.826%)	797 (11.588%)	1760 (31.987%)	2408 (53.044%)	2448 (66.050%)	
Smoking status, n (%)						< 0.0001
Former	3632 (21.078%)	930 (19.496%)	951 (20.741%)	909 (21.171%)	842 (24.049%)	
Never	11613 (59.816%)	3564 (64.597%)	3135 (60.724%)	2791 (58.982%)	2123 (51.648%)	
Now	3336 (19.106%)	816 (15.907%)	846 (18.534%)	817 (19.847%)	857 (24.303%)	
Alcohol use, n (%)						< 0.0001
Former	3105 (14.301%)	602 (9.431%)	798 (13.680%)	852 (16.186%)	853 (20.971%)	
Heavy	2999 (17.526%)	955 (18.745%)	832 (18.908%)	679 (16.285%)	533 (15.037%)	
Mild/moderate	8809 (53.269%)	2888 (59.742%)	2383 (52.979%)	1991 (50.239%)	1547 (46.603%)	
Never	3668 (14.903%)	865 (12.082%)	919 (14.434%)	995 (17.290%)	889 (17.389%)	
CHD, n (%)						< 0.0001
No	18092 (97.704%)	5240 (98.841%)	4837 (98.407%)	4369 (97.155%)	3646 (95.486%)	
Yes	489 (2.296%)	70 (1.159%)	95 (1.593%)	148 (2.845%)	176 (4.514%)	
DM, n (%)						< 0.0001
No	15554 (88.116%)	5021 (96.470%)	4358 (91.848%)	3561 (84.573%)	2614 (73.279%)	
Yes	3027 (11.884%)	289 (3.530%)	574 (8.152%)	956 (15.427%)	1208 (26.721%)	
Hypertension, n (%)						< 0.0001
No	10762 (63.558%)	3873 (78.000%)	2966 (66.207%)	2252 (54.605%)	1671 (46.621%)	
Yes	7819 (36.442%)	1437 (22.000%)	1966 (33.793%)	2265 (45.395%)	2151 (53.379%)	
Hyperlipidemia, n (%)						< 0.0001
No	4895 (28.202%)	2830 (54.113%)	1477 (30.237%)	513 (11.757%)	75 (2.104%)	
Yes	13686 (71.798%)	2480 (45.887%)	3455 (69.763%)	4004 (88.243%)	3747 (97.896%)	
Height, cm	162.158 ± 0.083	163.341 ± 0.125	162.083 ± 0.139	161.503 ± 0.132	161.092 ± 0.161	< 0.0001
Waist, cm	95.665 ± 0.209	84.132 ± 0.205	94.480 ± 0.255	102.403 ± 0.326	108.365 ± 0.321	< 0.0001
TC, mg/dL	199.007 ± 0.485	190.175 ± 0.667	196.524 ± 0.794	202.060 ± 0.819	213.448 ± 1.038	< 0.0001
TG, mg/dL	133.174 ± 1.162	65.812 ± 0.338	103.610 ± 0.554	147.783 ± 0.809	268.570 ± 3.253	< 0.0001
HDL, mg/dL	58.423 ± 0.240	71.994 ± 0.351	59.535 ± 0.229	51.648 ± 0.200	42.519 ± 0.209	< 0.0001
eGFR, mL/min/1.73 m^2^	94.139 ± 0.331	98.626 ± 0.417	94.436 ± 0.438	91.331 ± 0.518	89.652 ± 0.504	< 0.0001

Continuous variables are expressed as weighted means (± SE) and categorical variables as unweighted frequencies (weighted percentages).

### Gender differences in the relationship between CMI and outcomes

**Figure 2:**
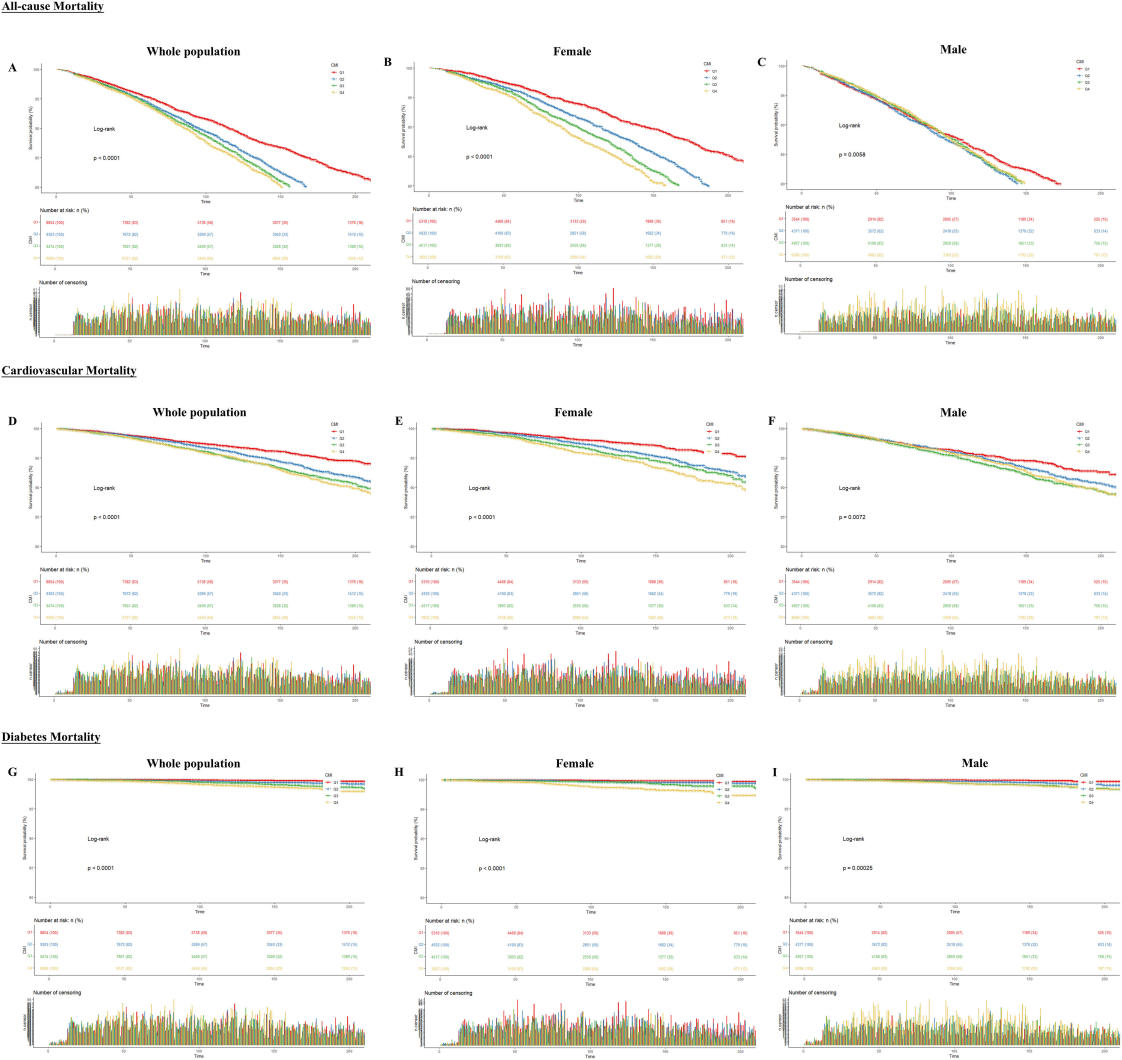
Kaplan-Meier curves between CMI and all-cause and specific mortality rates. The X-axis indicates follow-up time (months); the Y-axis indicates survival probability. **(A)** All-cause mortality for the whole population **(B)** All-cause mortality for females **(C)** All-cause mortality for males **(D)** Cardiovascular mortality for the whole population **(E)** Cardiovascular mortality for females **(F)** Cardiovascular mortality for males **(G)** Diabetes mortality for the whole population **(H)** Diabetes mortality for females **(I)** Diabetes mortality for males.

Throughout the monitoring phase, participants experienced 5600 deaths due to any cause, comprising 1765 from cardiovascular causes and 200 from diabetes. As shown in **Figure 2**, we performed Kaplan-Meier analysis. The results showed that CMI differed significantly in predicting all-cause, cardiovascular, and diabetes mortality between Q1-Q4 groups in the total population, females and males. All three mortality rates showed an increasing trend with increasing follow-up time, and we found no gender differences. Nevertheless, the differences were more pronounced in the female than in the male group.

**Tables 3**–**5** show the results of the COX regression analyses between CMI and outcomes, respectively. When CMI was a continuous variable, CMI in model 3 was significantly correlated with increased mortality rates due to all causes, cardiovascular and diabetes in the whole population (all-cause: HR: 1.03, 95%CI: 1.02-1.05, P<0.0001; cardiovascular: HR: 1.04, 95%CI: 1.02-1.05, P <0.0001; diabetes: HR: 1.11, 95%CI: 1.09-1.14, P<0.0001) and participants of both genders (all-cause: male: HR: 1.03, 95%CI: 1.01-1.04, P<0.001; female: HR: 1.05, 95%CI: 1.02-1.07, P<0.001. cardiovascular: male: HR: 1.03, 95%CI: 1.01-1.05, P=0.01; female: HR: 1.05, 95%CI: 1.03-1.08, P<0.0001. diabetes: male: HR: 1.09, 95%CI: 1.06-1.12, P<0.0001; female: HR: 1.15, 95%CI: 1.09-1.22, P<0.0001). As a categorical variable based on quartiles, CMI demonstrated a notable rise in mortality rates for all causes, cardiovascular disease, and diabetes in Q4 compared with Q1 in the whole population (all-cause: P for trend=0.005; cardiovascular: P for trend<0.001; diabetes: P for trend<0.001) and female participants (all-cause: P for trend=0.03; cardiovascular: P for trend= 0.005; diabetes: P for trend=0.002). In contrast, male participants had no significant difference (all-cause: P for trend=0.06; cardiovascular: P for trend=0.06; diabetes: P for trend=0.09).

**Table 3:** Models of COX regression for CMI and all-cause mortality.

	Model 1 HR (95% CI)	P	Model 2 HR (95% CI)	P	Model 3 HR (95% CI)	P
Whole population						
CMI	1.03 (1.02,1.03)	<0.0001	1.03 (1.02,1.04)	<0.0001	1.03 (1.02,1.05)	<0.0001
Categories						
Q1	Reference		Reference		Reference	
Q2	1.38 (1.23,1.55)	<0.0001	1.01 (0.91,1.12)	0.87	0.97 (0.86,1.08)	0.57
Q3	1.59 (1.45,1.75)	<0.0001	1.04 (0.94,1.14)	0.50	0.98 (0.88,1.08)	0.64
Q4	1.82 (1.62,2.05)	<0.0001	1.28 (1.14,1.43)	<0.0001	1.16 (1.03,1.30)	0.01
P for trend		<0.0001		<0.0001		0.005
Male						
CMI	1.01 (0.99,1.02)	0.28	1.02 (1.01,1.04)	0.001	1.03 (1.01,1.04)	<0.001
Categories						
Q1	Reference		Reference		Reference	
Q2	1.18 (1.00,1.38)	0.05	0.96 (0.82,1.12)	0.59	0.96 (0.82,1.14)	0.66
Q3	1.21 (1.05,1.40)	0.01	0.94 (0.82,1.09)	0.42	0.94 (0.81,1.09)	0.43
Q4	1.31 (1.13,1.52)	<0.001	1.14 (0.98,1.33)	0.09	1.12 (0.96,1.31)	0.14
P for trend		<0.001		0.03		0.06
Female						
CMI	1.05 (1.03,1.06)	<0.0001	1.05 (1.03,1.07)	<0.0001	1.05 (1.02,1.07)	<0.001
Categories						
Q1	Reference		Reference		Reference	
Q2	1.51 (1.29,1.78)	<0.0001	1.04 (0.89,1.21)	0.65	0.97 (0.83,1.14)	0.70
Q3	1.95 (1.70,2.23)	<0.0001	1.11 (0.95,1.30)	0.17	1.01 (0.86,1.18)	0.93
Q4	2.43 (2.07,2.84)	<0.0001	1.41 (1.20,1.66)	<0.0001	1.20 (1.02,1.41)	0.02
P for trend		<0.0001		<0.001		0.03

Model 1: non-adjusted.

Model 2: adjusted for age, gender, and race.

Model 3: Whole population adjusted for age, gender, race, marital, smoking, alcohol, eGFR, and TC. Male and female people adjusted for age, race, marital, smoking, alcohol, eGFR, and TC.

eGFR, estimated glomerular filtration rate; TC, total cholesterol.

HR, hazard ratios; CI, confidence interval.

**Table 4:** Models of COX regression for CMI and cardiovascular mortality.

	Model 1 HR (95% CI)	P	Model 2 HR (95% CI)	P	Model 3 HR (95% CI)	P
Whole population						
CMI	1.03 (1.02,1.03)	<0.0001	1.04 (1.02,1.05)	<0.0001	1.04 (1.02,1.05)	<0.0001
Categories						
Q1	Reference		Reference		Reference	
Q2	1.54 (1.27,1.87)	<0.0001	1.08 (0.90,1.31)	0.41	1.03 (0.85,1.25)	0.74
Q3	1.78 (1.50,2.12)	<0.0001	1.12 (0.93,1.34)	0.23	1.04 (0.86,1.26)	0.69
Q4	2.17 (1.81,2.61)	<0.0001	1.51 (1.26,1.82)	<0.0001	1.36 (1.12,1.64)	0.002
p for trend		<0.0001		<0.0001		<0.001
Male						
CMI	1.00 (0.99,1.02)	0.73	1.03 (1.01,1.04)	0.01	1.03 (1.01,1.05)	0.01
Categories						
Q1	Reference		Reference		Reference	
Q2	1.18 (0.88,1.57)	0.26	0.94 (0.71,1.26)	0.70	0.93 (0.69,1.26)	0.63
Q3	1.30 (1.02,1.64)	0.03	1.00 (0.78,1.29)	0.99	0.98 (0.75,1.28)	0.88
Q4	1.42 (1.13,1.80)	0.003	1.27 (0.98,1.65)	0.07	1.22 (0.93,1.59)	0.15
p for trend		0.002		0.03		0.06
Female						
CMI	1.05 (1.03,1.06)	<0.0001	1.06 (1.04,1.08)	<0.0001	1.05 (1.03,1.08)	<0.0001
Categories						
Q1	Reference		Reference		Reference	
Q2	1.88 (1.46,2.42)	<0.0001	1.19 (0.93,1.53)	0.16	1.12 (0.88,1.44)	0.35
Q3	2.24 (1.74,2.87)	<0.0001	1.20 (0.92,1.57)	0.18	1.08 (0.82,1.41)	0.58
Q4	3.15 (2.44,4.06)	<0.0001	1.75 (1.35,2.26)	<0.0001	1.49 (1.15,1.93)	0.002
p for trend		<0.0001		<0.001		0.005

Model 1: non-adjusted.

Model 2: adjusted for age, gender, and race.

Model 3: Whole population adjusted for age, gender, race, marital, smoking, alcohol, eGFR, and TC. Male and female people adjusted for age, race, marital, smoking, alcohol, eGFR, and TC.

eGFR, estimated glomerular filtration rate; TC, total cholesterol.

HR, hazard ratios; CI, confidence interval.

**Table 5:** Models of COX regression for CMI and diabetes mortality.

	Model 1 HR (95% CI)	P	Model 2 HR (95% CI)	P	Model 3 HR (95% CI)	P
Whole population						
CMI	1.06 (1.04,1.08)	<0.0001	1.08 (1.06,1.09)	<0.0001	1.11 (1.09,1.14)	<0.0001
Categories						
Q1	Reference		Reference		Reference	
Q2	2.27 (0.93, 5.53)	0.07	1.76 (0.73, 4.28)	0.21	1.59 (0.64,3.93)	0.32
Q3	3.90 (1.70, 8.96)	0.001	2.77 (1.21, 6.31)	0.02	2.27 (0.95,5.41)	0.06
Q4	5.92 (2.62,13.39)	<0.0001	4.57 (1.99,10.47)	<0.001	3.60 (1.48,8.76)	0.005
p for trend		<0.0001		<0.0001		<0.001
Male						
CMI	1.05 (1.03,1.06)	<0.0001	1.05 (1.03,1.08)	<0.0001	1.09 (1.06,1.12)	<0.0001
Categories						
Q1	Reference		Reference		Reference	
Q2	2.74 (0.58,12.89)	0.20	2.28 (0.48,10.86)	0.30	2.21 (0.46,10.75)	0.32
Q3	4.42 (1.02,19.17)	0.05	3.49 (0.80,15.18)	0.10	3.22 (0.68,15.20)	0.14
Q4	3.75 (0.84,16.66)	0.08	3.12 (0.69,14.03)	0.14	2.92 (0.61,14.01)	0.18
p for trend		0.02		0.05		0.09
Female						
CMI	1.07 (1.05,1.10)	<0.0001	1.11 (1.06,1.16)	<0.0001	1.15 (1.09, 1.22)	<0.0001
Categories						
Q1	Reference		Reference		Reference	
Q2	1.88 (0.62, 5.68)	0.26	1.37 (0.47, 4.00)	0.57	1.21 (0.40, 3.66)	0.73
Q3	3.26 (1.14, 9.31)	0.03	2.02 (0.72, 5.61)	0.18	1.60 (0.54, 4.73)	0.39
Q4	9.56 (3.65,25.05)	<0.0001	6.10 (2.33,15.95)	<0.001	4.60 (1.58,13.38)	0.01
p for trend		<0.0001		<0.001		0.002

Model 1: non-adjusted.

Model 2: adjusted for age, gender, and race.

Model 3: Whole population adjusted for age, gender, race, marital, smoking, alcohol, eGFR, and TC. Male and female people adjusted for age, race, marital, smoking, alcohol, eGFR, and TC.

eGFR, estimated glomerular filtration rate; TC, total cholesterol.

HR, hazard ratios; CI, confidence interval.

### RCS analysis

**Figure 3:**
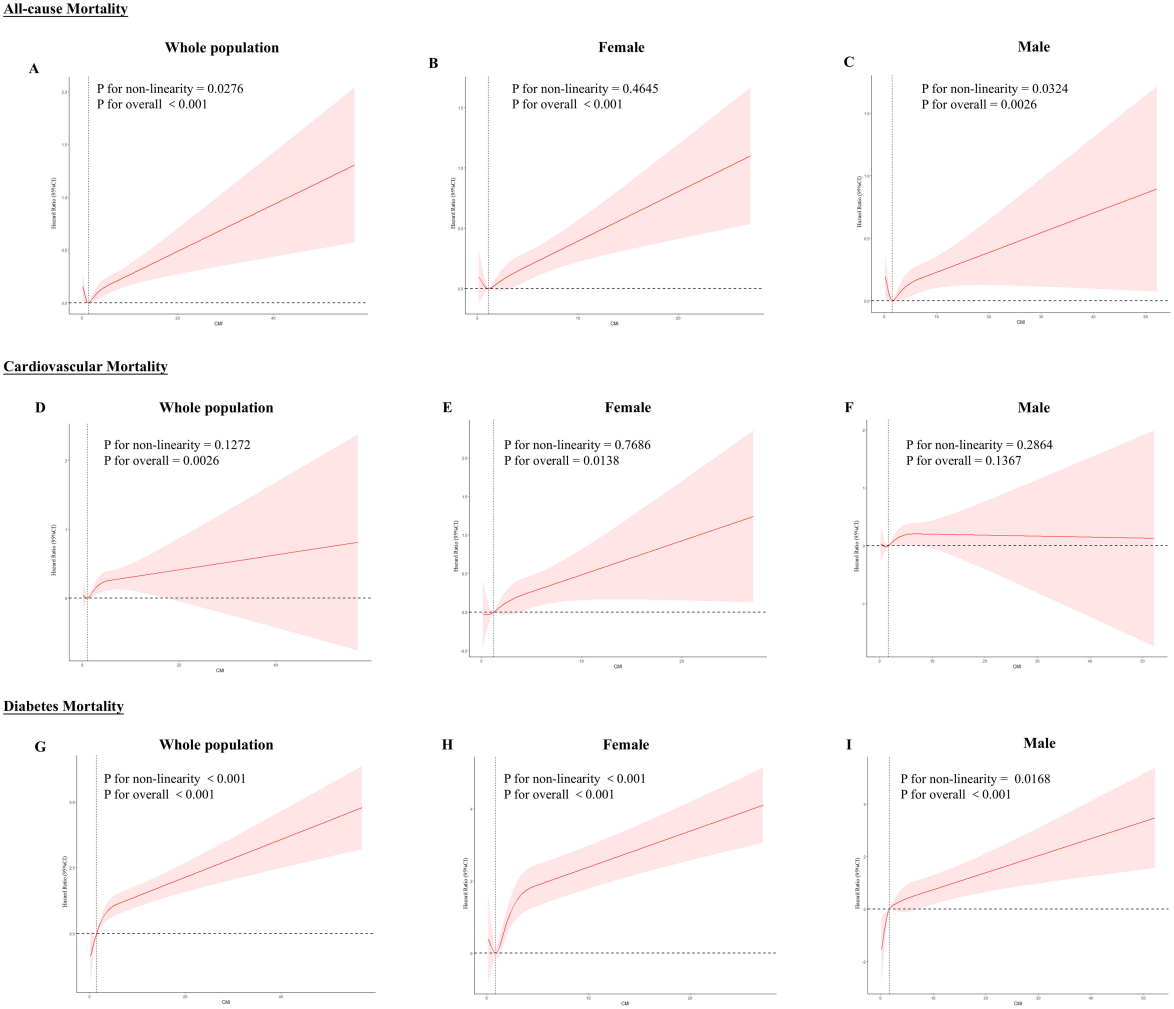
RCS curves between CMI and all-cause and specific mortality rates. Red lines represent the estimated hazard ratio, and the shaded red area corresponds to the 95% confidence intervals. **(A)** All-cause mortality for the whole population **(B)** All-cause mortality for females **(C)** All-cause mortality for males **(D)** Cardiovascular mortality for the whole population **(E)** Cardiovascular mortality for females **(F)** Cardiovascular mortality for males **(G)** Diabetes mortality for the whole population **(H)** Diabetes mortality for females **(I)** Diabetes mortality for males.

We assessed the RCS using model 3 to investigate further the correlation between CMI and mortality rates (**Figure 3**), which showed that in the total population, CMI and all-cause mortality were nonlinearly related (P for nonlinear = 0.0276), as well as diabetes mortality (P for nonlinearity = 0.001). Cardiovascular mortality was linearly related (P for nonlinear = 0.1272). All three mortality rates increased with increasing CMI. Across sexes, females showed a linear response relationship in all-cause mortality (P for nonlinear = 0.4645) and cardiovascular mortality (P for nonlinear = 0.7686) and a nonlinear response relationship in diabetes mortality (P for nonlinear < 0.001); males showed a nonlinear response relationship in all-cause mortality (P for nonlinear = 0.0324) and diabetes mortality (P for nonlinear = 0.0168), but there was no statistically significant relationship with cardiovascular mortality (P for overall = 0.1367).

## Discussion

Using NHANES data, we conducted a cohort study of 37,539 participants. The results showed significant gender differences between CMI and all-cause, cardiovascular, and diabetes mortality among the general adult population in the United States. In women, we found that CMI independently predicted mortality from all causes, cardiovascular diseases, and diabetes, but this association did not hold for men. Moreover, we further explored the trend changes by Kaplan-Meier and RCS curves. It is the first study to address gender differences in the correlation between CMI and mortality in the general adult population.

Researchers initially used CMI, a relatively new measure of obesity and lipid levels, to screen for diabetes and obesity (9). Subsequently, many related studies have shown a correlation between CMI and an increased risk of developing diabetes, with Zha et al. (30) finding a nonlinear positive relationship between CMI and diabetes risk in Japanese adults and Qiu et al. (31) finding the same positive association in a study of middle-aged and older adults in China. In addition, studies have also found associations between CMI and a variety of metabolic disorders, such as CVD. For example, Cai et al. (32) found that CMI was positively associated with the risk of new-onset CVD in patients with hypertension and obstructive sleep apnea. The above studies suggest that CMI can not only be used to assess diabetes but is also significantly associated with metabolism-related diseases. It is consistent with this study’s finding that CMI is significantly and positively associated with all-cause, cardiovascular, and diabetes mortality in the U.S. adult population.

Our study population was the general adult population, and we further analyzed that there was a significant correlation between CMI and all-cause mortality, cardiovascular mortality, and diabetes mortality in females. We tried to explain the reasons for the emergence of this gender difference.

Based on previous reports, it appears that cardiovascular and diabetic disease risk differs by gender. In older American populations, white men have a higher risk of obesity, whereas white women have a higher risk of hypertension and diabetes (33). Similarly, in Asian populations, research in a Japanese population showed a stronger relationship between obesity and cardiometabolic risk factors in women (34) and a greater cardiometabolic risk of glucose and dyslipidemia in women than in men (35). Shi et al. (36) found that higher AIP levels in women significantly increase the risk of developing prediabetes and diabetes. In women, impaired glucose tolerance (IGT) is more prevalent than in men, and obesity-associated type 2 diabetes mellitus is associated with significant gender and regional differences (37). The above findings suggest that gender differences in cardiovascular-related and diabetes-related diseases are more common among populations in various regions. Few studies have now reported sex differences between CMI and cardiovascular and diabetes-related diseases. Elevated CMI is associated with eccentric and centripetal left ventricular hypertrophy in females compared with males (38). It suggests that CMI is likely to be a marker for explaining the adverse cardiovascular effects of central obesity (39, 40). Moreover, the correlation between CMI and diabetes mellitus is more pronounced among female patients (9, 15). The above result is in line with the findings in the current study.

Gender differences in CMI reflecting adverse cardiometabolic outcomes may be related to the characteristics of body fat distribution in different gender populations. The specific mechanisms may be related to sex hormones, adipocyte characteristics, and the adipose microenvironment. Premenopausal women usually have more subcutaneous adipose tissue (SAT). In contrast, men have more trunk and visceral adipose tissue (VAT), and this characteristic shifts after menopause, with an increase in the distribution of trunk and VAT, the development of centripetal obesity, and an increase in waist circumference in women compared with that in men, which dramatically increases the risk of diabetes mellitus and cardiovascular disease in women (41–44). The CMI measures the lipid profile of the body, and the CMI is an indicator of cardiometabolic disease risk in women. While CMI is a comprehensive indicator for detecting dyslipidemia and abdominal obesity, it can reflect VAT distribution well. It appears to be more relevant to women in predicting adverse outcomes in cardiometabolic-related diseases. At the same time, we should also be concerned about the impact of obesity on adverse outcomes in other pre-existing conditions. The clinical phenotype of obesity is similar to that of hypertrophic cardiomyopathy (HCM) (45). Relevant studies have shown that among patients with HCM, women are older than men and have a higher risk of HCM-related complications and death (46, 47). Obesity is also a significant risk factor for increased rates of adverse events in patients with conditions such as stroke (48), thromboembolic disease (49), and atrial fibrillation (50).

In addition, an imbalance in female sex hormones significantly increases the risk of cardiometabolic issues. Sex hormones may influence the distribution of adipose molecules. Estrogen may affect fat distribution through its effect on adrenergic receptor distribution in long-lasting adipocytes. Testosterone may also play a specific role in adipose tissue distribution through its impact on lipoprotein lipase (LPL) activity and rate-limiting fatty acid uptake (51). Sex hormones also influence adipose cycling factors (e.g., leptin and lipocalin), which are directly related to the accumulation of SAT (52). The absence of positive results in males in this study may be related to the role of androgens. Androgens are involved in regulating lipid metabolism and may inhibit fat deposition (53). Therefore, high androgen levels in men may protect against lipid metabolism-related diseases.

Intrinsic properties of adipocytes may also account for gender differences in fat distribution and function. Adipocytes from SAT and VAT have different physiological properties, with STA adipocytes undergoing hyperplasia and hypertrophy, whereas VAT adipocytes experience hypertrophy only (54). Researchers have linked this intracellular difference to distinct patterns of gene expression. In addition to the intrinsic properties of adipocytes, differences in the extrinsic microenvironment and extracellular matrix can also lead to gender differences in adipose distribution, which is related to the regulation of the activation of adipocyte precursors that drive adipocyte proliferation (55).

In summary, the results of our study indicate that CMI is significantly linked to an increased risk of all-cause mortality, cardiovascular mortality, and diabetes mortality among adult women. It suggests that we may be able to judge the prognostic risk by the change of CMI value in clinical practice, especially for patients with cardiac disease, diabetes, and other underlying diseases. It is also important to pay attention to the influence of gender differences on disease prognosis. We recommended that adult women with high CMI values regularly engage in moderate exercise and pay attention to the management of lipids and waist circumference in order to reduce the risk of adverse prognosis of cardiometabolic-related diseases (56). More generally, it is worthwhile for medical personnel to consider whether there is a need to formulate different treatment standards and protocols according to gender in clinical diagnosis and treatment. At the same time, it is also necessary to pay attention to the screening of the relevant population. Clinical staff can apply new electrocardiography, echocardiography, and cardiac CT imaging to better screen high-risk groups (57–59) and reduce or delay the occurrence of cardiometabolic-related adverse outcomes.

However, this study has some limitations: (i) Since this study only examined the U.S. population, more studies on other races are required for generalizability. (ii) Human reporting factors may affect the accuracy of NHANES data on causes of death, population classification, and death certificates. (iii) We considered potential confounders, but other factors may remain unconsidered.

## Conclusion

In our study, we found that CMI was significantly positively associated with all-cause mortality, cardiovascular mortality, and diabetes mortality in adult females in the United States but not in males. According to the findings, it is plausible that CMI can be employed as a predictive indicator for mortality, particularly in relation to all-cause mortality, cardiovascular-specific mortality, and diabetes mortality among adult females.

## Data Availability

The original contributions presented in the study are included in the article/**Supplementary Material**. Further inquiries can be directed to the corresponding author.
