# Supplementary material for: Gender differences in the relationship between cardiometabolic index and all-cause and specific mortality in the United States adults: a national study
Source: Front Endocrinol (Lausanne). 2025 Feb 17;16:1525815. doi: 10.3389/fendo.2025.1525815 (PMC11872713; doi:10.3389/fendo.2025.1525815)
Supplement: Supplementary file 1 [file Table1.docx]

**Table 1S** Baseline characteristics of participants by survival status

| **Variables** | **Survival Status** | | | **P-value** |
| --- | --- | --- | --- | --- |
|  | **Total**  **(n=37539)** | **Survival**  **(n=31939)** | **Non-survival**  **(n=5600)** |  |
| CMI | 2.112±0.022 | 2.083±0.024 | 2.349±0.052 | < 0.0001 |
| Age (years) | 46.891±0.198 | 44.654±0.198 | 65.875±0.312 | < 0.0001 |
| Gender, n (%) |  |  |  | < 0.0001 |
| Female | 18581(50.472%) | 16198(50.865%) | 2383(47.135%) |  |
| Male | 18958(49.528%) | 15741(49.135%) | 3217(52.865%) |  |
| Race, n (%) |  |  |  | < 0.0001 |
| Mexican American | 6432(7.739%) | 5731(8.229%) | 701(3.581%) |  |
| Non-Hispanic Black | 7400(9.972%) | 6368(9.980%) | 1032(9.902%) |  |
| Non-Hispanic White | 17614(70.942%) | 14138(69.857%) | 3476(80.148%) |  |
| Other Race | 3144(6.161%) | 2983(6.496%) | 161(3.314%) |  |
| Others Hispanic | 2949(5.186%) | 2719(5.438%) | 230(3.055%) |  |
| Marital status, n (%) |  |  |  | < 0.0001 |
| Divorced | 4025(10.298%) | 3360(10.109%) | 665(11.907%) |  |
| Living with partner | 2795(7.494%) | 2627(7.930%) | 168(3.797%) |  |
| Married | 20077(57.145%) | 17235(57.634%) | 2842(52.994%) |  |
| Never married | 6439(17.196%) | 6045(18.314%) | 394(7.706%) |  |
| Separated | 1211(2.384%) | 1056(2.401%) | 155(2.242%) |  |
| Widowed | 2992(5.483%) | 1616(3.612%) | 1376(21.355%) |  |
| Education, n (%) |  |  |  | < 0.0001 |
| Less than 9th grade | 4052(5.103%) | 2964(4.296%) | 1088(11.952%) |  |
| 9-11th grade | 5370(10.715%) | 4315(9.890%) | 1055(17.720%) |  |
| High school graduate | 8702(23.894%) | 7267(23.358%) | 1435(28.442%) |  |
| Some College | 10961(31.496%) | 9710(32.171%) | 1251(25.768%) |  |
| College graduate or above | 8454(28.792%) | 7683(30.285%) | 771(16.119%) |  |
| PIR |  |  |  | < 0.0001 |
| <1.30 | 11096(20.182%) | 9190(19.415%) | 1906(26.687%) |  |
| 1.30-3.50 | 14283(35.581%) | 11825(34.559%) | 2458(44.255%) |  |
| ≥3.50 | 12160(44.237%) | 10924(46.027%) | 1236(29.058%) |  |
| BMI, kg/m^2^ |  |  |  | 0.243 |
| <25 | 11072(31.052%) | 9354(31.082%) | 1718(30.803%) |  |
| 25-30 | 12784(33.488%) | 10741(33.335%) | 2043(34.784%) |  |
| ≥30 | 13683(35.460%) | 11844(35.584%) | 1839(34.413%) |  |
| Smoking status, n (%) |  |  |  | < 0.0001 |
| Former | 9486(25.091%) | 7263(23.613%) | 2223(37.627%) |  |
| Never | 20035(53.372%) | 17835(55.179%) | 2200(38.050%) |  |
| Now | 8018(21.537%) | 6841(21.208%) | 1177(24.324%) |  |
| Alcohol use, n (%) |  |  |  | < 0.0001 |
| Former | 6467(14.143%) | 4582(12.118%) | 1885(31.327%) |  |
| Heavy | 7597(21.479%) | 7006(22.626%) | 591(11.745%) |  |
| Mild/moderate | 18366(53.632%) | 16131(54.959%) | 2235(42.374%) |  |
| Never | 5109(10.746%) | 4220(10.297%) | 889(14.554%) |  |
| CHD, n (%) |  |  |  | < 0.0001 |
| No | 35970(96.582%) | 31084(97.662%) | 4886(87.418%) |  |
| Yes | 1569(3.418%) | 855(2.338%) | 714(12.582%) |  |
| DM, n (%) |  |  |  | < 0.0001 |
| No | 31180(87.597%) | 27323(89.365%) | 3857(72.596%) |  |
| Yes | 6359(12.403%) | 4616(10.635%) | 1743(27.404%) |  |
| Hypertension, n (%) |  |  |  | < 0.0001 |
| No | 21734(63.221%) | 20092(66.839%) | 1642(32.529%) |  |
| Yes | 15805(36.779%) | 11847(33.161%) | 3958(67.471%) |  |
| Hyperlipidemia, n (%) |  |  |  | < 0.0001 |
| No | 10386(28.809%) | 9324(30.086%) | 1062(17.977%) |  |
| Yes | 27153(71.191%) | 22615(69.914%) | 4538(82.023%) |  |
| Height, cm | 169.073±0.081 | 169.280±0.085 | 167.318±0.171 | < 0.0001 |
| Waist, cm | 98.484±0.168 | 98.112±0.180 | 101.634±0.266 | < 0.0001 |
| TC, mg/dL | 196.596±0.403 | 196.379±0.422 | 198.436±0.811 | 0.016 |
| TG, mg/dL | 150.864±1.119 | 149.548±1.226 | 162.028±2.251 | < 0.0001 |
| HDL, mg/dL | 53.087±0.173 | 53.101±0.183 | 52.965±0.351 | 0.717 |
| eGFR, mL/min/1.73 m^2^ | 94.016±0.263 | 96.254±0.270 | 75.030±0.436 | < 0.0001 |

Continuous variables are expressed as weighted means (±SE) and categorical variables as unweighted frequencies (weighted percentages).
